# Young Investigator Award 2026: Extent and incidence of pseudo-worsening of kidney function due to oral antitumor therapeutics in the AMBORA cohort: an analysis of real-world data

**DOI:** 10.1007/s00210-026-05651-9

**Published:** 2026-07-01

**Authors:** Michael I. Sponfeldner

**Affiliations:** Institute of Experimental and Clinical Pharmacology and Toxicology, Friedrich-Alexander-Universität Erlangen-Nürnberg, Erlangen, Germany

**Keywords:** Cancers, Kidney, Oncology

Oral antitumor therapeutics (OATs) are part of numerous therapy regimens in the treatment of solid tumors and hemato-oncological malignancies, with more than 100 different OATs approved until today (National Cancer Institute n.d.; Aisner  2007). Especially in oncology and during treatment with OATs, close monitoring of kidney function is crucial, since renal insufficiency is common among cancer patients due to comorbidities, advanced age, or certain nephrotoxic antitumor drugs (Sandhu et al. 2022; Malyszko et al. 2020).

Kidney function is typically assessed by measurement of serum creatinine concentrations (SCr) and subsequent calculation of estimated glomerular filtration rate (eGFR_SCr_) (Inker et al. 2021; Levey et al. 2009; Levey et al. 1999). Next to passive glomerular filtration, creatinine is actively secreted into urine by renal transport proteins such as organic cation transporter 2 or multidrug and toxin extrusion proteins 1 and 2-K (Levey et al. 1988; Urakami et al. 2004; Tanihara et al. 2007). It has been shown that several OATs inhibit those renal creatinine transporters, leading to increases in SCr and decreases in eGFR_SCr_ without inducing kidney injury, as shown by other methods of kidney function assessment such as measurement of exogenous filtration markers such as iohexol or eGFR based on cystatin C (eGFR_CysC_) (Buijs et al. 2025; Bruin et al. 2021; Chappell et al. 2019; Chen et al. 2024; Fromm et al. 2025; Sponfeldner et al. 2024; Sy-Go et al. 2022; Vidal-Petiot et al. 2016; Zibetti et al. 2020). These OATs induce pseudo-worsening of kidney function. If unrecognized in clinical practice, this may pose patients at considerable risk for unnecessary OAT dose reductions, treatment interruptions, or discontinuations (Buijs et al. 2025). Since there still were limited data on incidence and extent of pseudo-worsening of kidney function during treatment with OATs in real-world clinical practice, our study aimed at a broad evaluation of pseudo-worsening of kidney function in patients suffering from various oncological diseases treated with a wide variety of OATs (Sponfeldner et al. 2026).

Therefore, we performed a retrospective cohort study, using data collected within the AMBORA cohort (Dürr et al. 2021; Cuba et al. 2024; Cuba et al. 2023). AMBORA is an intensified clinical pharmacological/pharmaceutical care program over 12 weeks after OAT initiation and longitudinally collects demographic and clinical data, adhering to standard operating procedures. Additionally, corresponding laboratory values (i.e., SCr, CysC, and eGFR_SCr_/eGFR_CysC_) as well as data on previous kidney transplantation (NTx), nephrotoxicity or other descriptions of kidney injury during the observation period were collected from electronic health records of the University Hospital Erlangen. Data were collected from OAT initiation plus 12 weeks. Patients were included, if sufficient clinical data were available such as a baseline SCr/eGFR_SCr_ measurement prior to OAT initiation (eGFR_SCr_ ≥ 30 ml/min) and ≥ 1 subsequent measurement of SCr/eGFR_SCr_ and excluded, if they, for example, previously underwent kidney transplantation or had a documentation of newly occurring kidney injury / nephrotoxicity during the observation period. Analyses were conducted for the three groups of patients receiving OAT: (1) with OAT likely causing pseudo-worsening of kidney function, (2) with proven pseudo-worsening of kidney function, and (3) as control group, receiving OAT which is unlikely to cause pseudo-worsening of kidney function. We assessed the maximum absolute and mean changes in SCr / eGFR_SCr_ within 30 days of OAT initiation, including stratified analyses for individual OAT. In addition, the incidence of concomitant CysC measurements, i.e., at OAT initiation and / or subsequent timepoints, within 30 days of OAT initiation was evaluated. Furthermore, the long-term changes in kidney function beyond the 30-day observation period were also analyzed.

Of 694 patients assessed for eligibility, 238 patients, treated with 38 different OATs likely causing or proven to cause pseudo-worsening of kidney function, were included. Among these, 114 patients (47.9%) received OAT (*n* = 18/38, 47.4%) proven to cause pseudo-worsening of kidney function. As a control group, an additional cohort of 67 patients treated with OAT unlikely to cause pseudo-worsening of kidney function was included.

Within the first 30 days of OAT initiation, a mean eGFR_SCr_ decline of 6.8 ml/min (± 14.5 ml/min) was observed in the 238-patient cohort treated with OAT likely causing or proven to cause pseudo-worsening of kidney function (*p* < 0.001). The 114-patient subgroup receiving OAT proven to cause pseudo-worsening of kidney function showed a mean decline in eGFR_SCr_ by − 11.1 ml/min ± 14.7 ml/min (*p* < 0.001). Within the control group of 67 patients treated with OAT unlikely to cause pseudo-worsening of kidney function, eGFR_SCr_ showed no significant decline. eGFR_SCr_ decreased ≥ 20 ml/min in 17.2% of the 238-patient cohort treated with OAT likely causing or proven to cause pseudo-worsening of kidney function. A patient treated with ribociclib experienced the largest observed decrease in eGFR_SCr_, i.e., − 52 ml/min.

The largest mean decreases in eGFR_SCr_ were observed in patients treated with niraparib (− 27.8 ml/min ± 18.9 ml/min, n.s.), followed by abemaciclib (− 20.6 ml/min ± 11.3 ml/min, *p* < 0.001), and ribociclib (− 20.5 ml/min ± 15.8 ml/min, *p* < 0.001). The incidence of decreases ≥ 10 ml/min in eGFR_SCr_ was in agreement with the extent of changes, with the highest incidence observed in patients treated with niraparib (100%, *n* = 3/3), followed by abemaciclib (86.7%, *n* = 13/15). When evaluating the long-term effect of OAT on SCr/eGFR_SCr_, no significant difference was observed (+ 0.9 ml/min, n.s.) when comparing the change from OAT initiation to within 30 days and from day 31 to week 12. CysC-based kidney function assessments were not performed in 95.8% of the 238 included patients.

Our study was the first to assess pseudo-worsening of kidney function across a broad range of OAT in a real-world cohort, without restricting analyses to specific OAT or cancer entities. Overall, we found that declines in eGFR_SCr_ are highly common at OAT initiation, particularly with OAT such as niraparib and cyclin-dependent kinase 4/6 inhibitors. The very limited use of creatinine-independent kidney function assessments (e.g., eGFR_CysC_) points to gaps in clinical awareness of treating physicians and may place patients at risk of avoidable, potentially serious medication errors. These findings highlight the need for education of oncologists on pseudo-worsening of kidney function. Moreover, prospective studies are warranted for OATs that showed significant declines in eGFR_SCr_ with a lack of confirmatory CysC data in literature, to be able to reliably differentiate pseudo-worsening from true kidney injury in routine practice.

I want to thank my colleagues of the AMBORA team as well as our collaborators at the Comprehensive Cancer Center Erlangen–EMN for their support during this project.

## Declarations

### Previous presentations:

Parts of this work were presented at the 10th German PharmTox Summit in Hannover (March 26-28, 2025, Hannover, Germany) and the EACPT 2025 Congress in Helsinki (June 28 – July 1, Helsinki, Finland).
